# Experiments on Animals

**Published:** 1920-06-12

**Authors:** Stephen Paget

**Affiliations:** Hon. Secretary Research Defence Society.


### THE EDITOR'S LETTER-BOX.

**Correspondence on all subjects is invited, but we cannot in any way be responsible for the opinions expressed by our correspondents, who must give their name and address as a guarantee of good faith, but not necessarily for publication. Correspondents are reminded that brevity of style and conciseness of statement greatly facilitate early insertion.**

### Experiments on Animals.

*To the Editor of* THE HOSPITAL.

SIR,—In view of the statements which are made against experiments on animals, I desire to call attention to the work of the Research Defence Society.

This Society was founded in 1908, with Lord Cromer as its President, to make generally known the facts as to experiments on animals in this country, and the regulations under which they are conducted; the immense importance of such experiments to the welfare of mankind; and the great saving of human and animal life and health which is already due to them. I shall always be glad to send some of the Society's publications to anybody who will send me a postcard; and I shall always be glad to answer questions, and to give exact references.

Only 3 or 4 per cent. of experiments on animals in this country involve any operation on any animal. No operation, more than the lancing of a vein under the skin, is allowed to be done on any animal, except under a proper anæsthetic. In the majority of these operation-cases the animal is put to death, then and there, while it is still under the anæsthetic. If the animal is allowed to recover from the anæsthetic and to be kept for observation, and if it is found in severe pain which is likely to continue, it must at once be killed under an anæsthetic.

No less than 96 or 97 per cent. of experiments on animals in this country are concerned with the study of germs, with the testing and standardising and making of curative and protective remedies, with the study of nutrition, with the diagnosis of obscure cases, with the study of heredity and of immunity, with the control of the national food supply and milk supply, with cases of imported disease, and so forth. None of these experiments involves operation; and many of them are absolutely painless.

These experiments mostly are made in the direct service of the national health and efficiency. Thus, in 1918, nearly 29,000 experiments were made on behalf of Government Departments and Public Health Authorities; and more than 37,000 for the testing, standardising, and preparing of remedies. The Home Office, the War Office, the Local Government Board, the Colonial Office, the Board of Agriculture, the London County Council, and the chief municipalities, all of them have made use of these methods. It is part of their duty to the nation. It is the business of the Government, and of public bodies, to keep anthrax and cholera and plague out of the country, to protect our workers in dangerous trades, to keep the germs of tubercle out of the national milk-jug, to ensure the proper strength of the nation's medicine, and the proper quality of the nation's food; and, in general, to safeguard the health and efficiency of the Navy, the Army, and the civil population. And it is to be noted that experiments on animals have led not only to the saving or safeguarding of the lives of men, women, and children, but also to the saving or safeguarding of the lives of horses, dogs, cattle, sheep, and swine.

I am old enough to remember the time when many of these great discoveries were not yet made. I am thankful to have seen the coming of them, thankful to have enjoyed the honour of knowing some of the men who made them. If anybody cares to read up this very important subject, and will write to me at 11 Chandos Street, Cavendish Square, London, W.1, I will gladly be of any use to him or to her.—Yours faithfully,

STEPHEN PAGET, F.R.C.S.,
Hon. Secretary Research Defence Society.

June 8, 1920.

